# The Virginia Tech Response

**DOI:** 10.1007/s10439-012-0660-y

**Published:** 2012-10-04

**Authors:** Steven Rowson, Stefan M. Duma

**Affiliations:** School of Biomedical Engineering and Sciences, Virginia Tech – Wake Forest University, Blacksburg, VA USA

We believe that Dr. Albert King’s letter regarding the STAR system paper^22^ is not scientifically based, factually incorrect, and illustrates that he does not understand the STAR system’s methodology. All of our research from Virginia Tech on this subject has been peer reviewed and published in open journals. In contrast, Dr. King bases his comments on a single reference to an internal graduate student thesis that has not been peer reviewed, has not been published in a journal, and which is not publicly available. This is not how the scientific process works.

While studying our comments, we ask the reader to keep in mind that all of this debate comes down to a very simple question: do you want to buy a helmet that reduces head acceleration? The STAR system is a complex experimental methodology that combines exposure and injury risk based on current scientific knowledge. Simply stated, it provides independent data to consumers to illustrate which helmets lower head acceleration.

## Dr. King’s Apparent Substantial Conflict of Interest

In order to understand the severity of Dr. King’s apparent conflict of interest, it is important to understand the entire timeline of our STAR paper relative to his letter to the editor. In May 2011, we published the STAR paper, after a 3-month open response period, and after full peer-review in the Annals of Biomedical Engineering (ABME). During this process, and for the entire next year, Dr. King did not submit any letters to ABME, nor did he submit any questions or comments to us regarding this paper.

Once he submitted his letter, we contacted Dr. King, but he refused to discuss his letter or any potential conflicts. In order to gain clarity, Virginia Tech was forced to file a Freedom of Information Act request with Wayne State University so that we could document the following timeline, contracts, and statements.

In May 2012, at the request of lawyers working for Xenith (a helmet manufacturer), a consulting company emailed experts around the country looking to retain anyone who would critique the STAR paper. This email stated that the Xenith helmet “did not fare well in the ratings.” They sent this email to Dr. King on May 9, 2012, and he responded to them within 2 h saying “it was a pleasure talking with you,” and he forwarded his CV. On May 17, 2012, Dr. King signed a contract with them entitled “Xenith LLC Consulting” which summarized the agreement that he would be paid $400/h and all reasonable expenses for his critique of the STAR paper.

Over the next several weeks, Dr. King filled out time sheets recording his consulting hours as he prepared his report for this company. On June 17, 2012, Dr. King produced a draft report on “Albert I. King Inc” letterhead entitled “Review of the STAR Report by Rowson and Duma.” A few days later on June 21, 2012, Dr. King submitted a letter to the Editor of ABME that was virtually identical to this draft report. Dr. King provided no disclosure of any conflicts of interest in this letter. The next day and while the letter was under review, Xenith began nationally distributing a report from Dr. King, also dated June 21, 2012, that was virtually identical to his letter to the Editor of ABME. This report also does not include any disclosure of any conflicts of interest.

On June 25, 2012, based on concerns raised about potential conflict of interest issues, Dr. King was specifically asked by the Editor of ABME to “provide any disclosures related to this work, in terms of retainers *etc*. from lawyers as well as companies.” Dr. King responded “I received no personal compensation from anyone for writing this letter to the editor.” For a second time, Dr. King did not disclose any relationship or potential conflict of interest, even when directly asked by the Editor.

On June 29, 2012, Dr. King was informed that ABME had a copy of the May 2012 email request from a company representing Xenith’s lawyers, as well as a copy of his June 21, 2012 report that Xenith was distributing and which was effectively identical to his letter. Because of potential copyright issues, Dr. King was asked to write a different letter. He was also asked to complete a much more detailed conflict of interest form. This form specifically stated that Dr. King must “report all sources of revenue paid (or promised to be paid)…from sources with relevance to the submitted work.” On July 19, 2012, Dr. King submitted his revised letter with a new story about his conflict of interest. In this disclosure Dr. King states that “I was originally asked by Xenith Corp to write a written review of the paper in question with remuneration.”

As the consulting story unfolded, we contacted Dr. King directly and informed him that we were aware of this serious conflict, and we asked for his explanation. Dr. King refused to respond to our questions; however, immediately following our inquiry, there were a series of emails between Dr. King, the consulting company, and Xenith. At one point Dr. King wrote Vin Ferrara, the CEO of Xenith, and stated that “I think we should cool it for a while.” These emails showed how Dr. King’s report changed from an “Albert I. King Inc.” report to a Wayne State University report when Dr. King stated that “it is now a public document and is likely to receive a lot of media attention.” Only then did Dr. King state that he did not want to be paid, but he added that “Mr. Ferrara can make a donation to Wayne State University.”

What is clear is that Dr. King agreed to write his report for payment from Xenith (or their representatives), and that Dr. King deceived the scientific process by intentionally withholding information that he had in fact been retained by Xenith (or their representatives) to write his report.

## Virginia Tech is Independent

We have no financial interest whatsoever with the HIT System or Riddell. We have never received any funding or royalties or promise of payment in any form from any helmet manufacturer. We submit that we are one of the only truly independent helmet research laboratories in the world. Our funding is unbiased and comes from the NIH, DOT, DOD, Toyota Central Research and Development Laboratories, and Virginia Tech, all of which have no interest in any helmet company.

## The HIT System is Well-Validated

Dr. King’s assertions that the HIT System is prone to large errors are not supported by any scientific peer reviewed data. Most importantly, the data collected in the referenced master’s thesis do not support his conclusions. In contrast, there is significant literature documenting the validation of the HIT System, which includes both laboratory and on-field testing.^1^,^5^,^20^,^23^

We summarize here the large body of research and development surrounding the HIT System and its validation, which has been employed on the field for the past 9 years at colleges and high schools across the country.^2^,^3^,^6^,^7^,^9^,^10^,^12^–^14^,^17^,^23^,^25^ The algorithms for the HIT system calculations were developed in part with funding from the National Institutes of Health. The algorithm was peer reviewed and published in the open literature.^5^ To date, over 2,000,000 head impacts have been recorded. These data have resulted in important coaching and policy changes from youth football to collegiate level practice schedules.^6^,^8^

**Figure 1:**
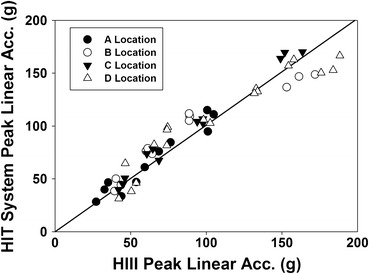
The NFL test data showing excellent correlation between the HIT system and the Hybrid III for linear acceleration over a range of velocities and impact locations^1^

Additionally, the NFL commissioned an independent study to evaluate the accuracy of the HIT System. This testing was performed independent of anyone from Virginia Tech. The NFL utilized a linear impactor and tested a wide range of impact velocities (4.4–11.2 m/s) and helmet locations. These locations and velocities were selected following detailed video review of on-field impacts to simulate the range of head contact sustained by NFL football players. The data were peer reviewed and published in ABME.^1^ Specifically, for the impacts on which the STAR system is based (front, back, top, and side), the linear acceleration correlation is high (*r* ^2^ = 0.90) between the HIT system and the Hybrid III (Fig. 1).

Regarding Dr. King’s comments on helmet fit methods and selection of helmet size for validation, in our research, and to our knowledge in all associated published research, the manufacturer guidelines for helmet fit were followed. A medium-sized Riddell Revolution football helmet was determined to best fit the Hybrid III head due to the unique geometry of the Hybrid III head. The 22.5″ measurement for head circumference is close to the borderline between medium and large sized helmets. Head circumference is merely a starting point for proper helmet fit, with size and shape of the head ultimately affecting best fit. While the Hybrid III circumference is slightly over the medium range, it’s other features (slim jaw line, no ears) dictate that a medium helmet should be used to achieve the manufacturer’s recommended fitting guidelines: The helmet should be (1) be approximately 1″ above the player’s eyebrows and (2) the skin of the forehead should move with the front pad. There should be no room for twisting of the helmet relative of the head.

Dr. King references an unpublished thesis (the Wayne State study) that claims large absolute errors when a large Riddell Revolution IQ helmet equipped with HIT System is tested. Without explanation, a proper fitting helmet on the Hybrid III head was not evaluated, but rather a loose fit and a tight fit with a large helmet. The rationale behind these loose and tight fit conditions are not well supported by the methods and data provided in the thesis, but more importantly, those data are not publicly available and have not been peer reviewed. Dr. King offers no valid data to support his contention that the “the contact pressures between the helmet and the head were way above the comfort level of human volunteer subjects who tested both the medium and large sized helmets.”

The unpublished Wayne State study performs impact tests at only one impact speed (9.7 m/s). This impact speed does not represent over 95% of the impacts players see in a game,^22^ and testing at one speed does not allow for reasonable correlations to be performed that could justify Dr. King’s statement. Dr. King’s assertion that the HIT System was validated against a “very tight fitting helmet” is not supported by any data in the literature. These examples show the lack of scientific rigor and lack of rationale used in Dr. King’s effort to undermine the credibility of the HIT System, and subsequently the STAR system.

## HIT System Measurements are Consistent with Other Studies

In one published study, the HIT System was used to measure head acceleration resulting from impact in 32 clinically diagnosed concussions on the field, with an average linear acceleration of 105 ± 27 g.^22^ Using an entirely different approach, the NFL conducted dummy experiments to reconstruct impacts associated with concussions observed in NFL games. That peer reviewed research found an average head acceleration of 98 g ± 28 g for the observed NFL concussions.^19^ The fact that these data sets are statistically identical is important independent validation.

Additionally, Virginia Tech has utilized a more advanced helmet impact system in parallel helmet studies for the past 5 years. This custom helmet system utilizes 12 accelerometers per helmet (compared to 6 in the HIT system). This system was also validated over a wide range of impact velocities and helmet locations.^20^ Its development and use was sponsored by the Department of Transportation and Toyota Motor Corporation in order to develop rotational acceleration head injury criteria.^15^,^23^,^26^,^27^ Unfortunately, each sensor costs over $10,000 and therefore it is not a feasible tool for wide-spread implementation. However, we have collected over 20,000 impacts with the custom system from over 40 players, and used these data^21^,^23^ to further demonstrate the ability of HIT System to accurately measure head accelerations in the field during a football game.

## Rotational Acceleration is Highly Correlated to Linear Acceleration

Dr. King’s assertion that the measurement of resultant linear head acceleration in the laboratory in the STAR system used to rate football helmet is “potentially harmful to players who may not be diagnosed with a concussion…” is not supported by any scientific data. His argument is not logical. Reducing head acceleration is an important objective in reducing injury risk and obviously will not have a negative effect on injury incidence. In fact, understanding and managing the energy transferred to the human body during impact loading is the basis of the injury biomechanics field.

Dr. King claims that it is inadequate to use a test based entirely on linear acceleration rather than one including rotational acceleration. He fails to understand that for the vast majority of impacts, if linear acceleration is reduced, rotational acceleration will also be reduced.^28^ The close correlation between linear acceleration and rotational acceleration in head impacts in football has been demonstrated in laboratory tests, in the NFL’s video reconstruction of head impacts,^19^ as well as the on-field HIT System data.^23^

## Linear Acceleration is a Good Predictor of Concussion

Linear acceleration has been shown to be very well correlated with concussion from head impacts in football.^12^,^16^,^19^ In fact, Dr. King’s own studies have reported the importance of linear acceleration. Dr. King participated in and analyzed reconstructions of concussive impacts in the NFL, and he found linear acceleration to be significantly correlated to the risk of concussion.^16^,^18^ Moreover, he found linear acceleration is a slightly better predictor of concussion than rotational acceleration alone. A follow-up study by Dr. King and colleagues in 2004 confirmed these findings.^30^ Nevertheless, we readily acknowledge that the complete understanding of the biomechanics of concussion has not yet been achieved. We are working to include rotational acceleration in the STAR system, but this can only be achieved through the rigorous scientific process of peer reviewed studies and publications.

## Concussions are not Diagnosed Using Head Acceleration

Importantly, it must be emphasized that concussions are diagnosed only by trained medical personnel based on the onset of symptoms. They are not diagnosed by head acceleration measurements as Dr. King contends. The HIT System and the STAR system are not designed to be diagnostic tools. Instead, they provide valuable additional information related to head impacts in football. The HIT System provides biomechanical data on head impacts experienced by players wearing instrumented helmets during play. The STAR system allows a biomechanical evaluation of the relative performance of adult football helmets in their ability to reduce the probability of concussion.^22^ Neither of these systems could be potentially harmful due to an effect on the diagnosis of concussion. Dr. King’s comments on the diagnosis of concussion are ill-informed.

## Validation of the STAR Evaluation System

To further illustrate the validity of the STAR system, we compare STAR values against available clinical data. The STAR system predicts the Riddell Revolution (4 star helmet) will result in a 54% reduction in risk of concussion compared to the Riddell VSR4 (1 star helmet). There are two clinical studies to compare this prediction. First, Collins *et al*.^4^ evaluated over 2000 high school players and found the Riddell Revolution significantly reduced the risk of concussion by 31% compared to other helmets.^4^ Second, our NIH funded research allowed us to collect clinical and biomechanical data that include athletes wearing both the Riddell Revolution and the Riddell VSR4.^24^ Over the last 9 years at Virginia Tech (2003–2011), we have instrumented our football players’ helmets with the HIT System. The resulting data allow us to investigate concussion rates by helmet type while accounting for each player’s exposure to head impact. For each helmet type, the number of impacts not resulting in diagnosed concussion was compared to the number of impacts that resulted in diagnosed concussion. Our data find a statistically significant 85% reduction in concussion risk for the Revolution helmets compared to the VSR4 helmets [χ^2^(1,153486) = 4.52, *p* = 0.03]. Our analysis includes 308 instrumented player-years with 153,486 head impacts and eliminates the previous criticisms of Collins *et al*.^4^ Helmet age was controlled, as each player had been provided with his own new helmet of either type. Also, the same physician made each concussion diagnosis throughout the 9 years. In summary, the STAR system predicts a 54% reduction in concussion risk between these two helmet models, and the two separate clinical studies show significant risk reductions of 31 and 85%.

Another study by the NFL examined the impact response of modern helmets using a linear impactor.^28^ A wide range of impact velocities and helmet locations were utilized and the data were summarized and provided to the NFL players. The rank order of helmets between the NFL linear impactor testing and the results from the STAR system are very similar. The only exception is the Xenith X1, which we ranked highly (4 stars), but the NFL found to be a poor performer. It is possible Xenith improved the design since the NFL tests were run, which was several years before our tests.

## Injury Risk Curves are Dependent on the Underlying Data

Dr. King fails to consider that an injury risk curve is not only dependent on the injury data, but also the full exposure data associated with no injury. Due to the challenging and time consuming testing methods used by Pellman *et al*.^19^, those researchers could only recreate 31 impacts in the laboratory, which resulted in a dataset that was biased toward concussive impacts because they could not account for all the impacts that a player experienced not resulting in diagnosed concussion. This means that the injury risk curves developed by Pellman *et al*.^19^ overestimate the risk of injury. A strength of HIT System is its ability to capture each impact that a player experiences, providing the full exposure data. Previous studies have shown that there are a great number of impacts that result in high head accelerations that do not result in diagnosed concussion.^12^,^21^ Unlike risk curves developed from laboratory reconstructions, the injury risk curve in the current manuscript considers the full exposure of all impacts, and is based on the data collected. Dr. King then makes an unsubstantiated leap to link the scientific findings of our published study to his opinion that the HIT System is prone to large errors. We cannot appreciate his motivation for this comment.

## NOCSAE Drop Tests

**Figure 2:**
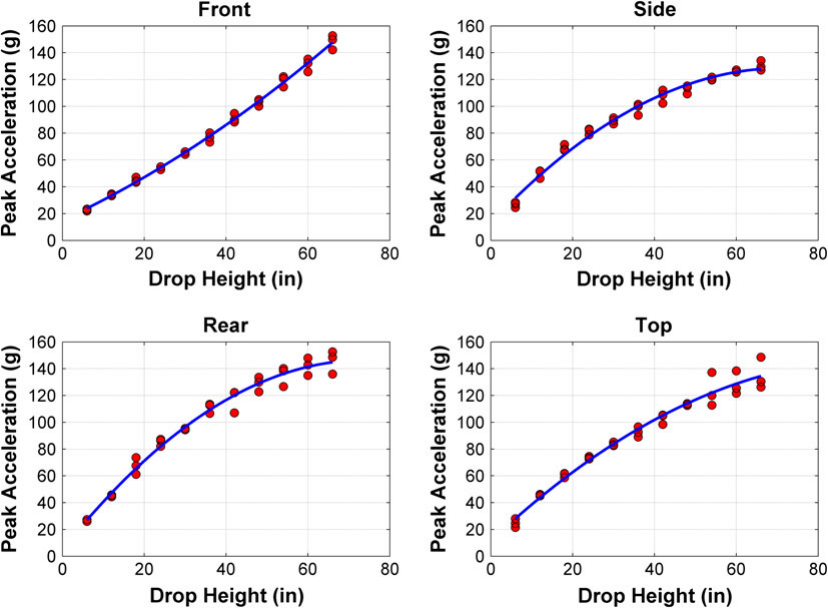
Peak acceleration data and quadratic regression lines for each impact locations from the 132 drop tests performed on 3 Riddell VSR4 helmets

To help Dr. King understand the findings of the STAR paper, we have provided plots below of the data from the 132 drop tests performed on 3 Riddell VSR4 helmets, with quadratic regression lines for each impact location (Fig. 2). As is standard scientific practice, we demonstrated the goodness of fit (*r* ^2^ = 0.99) and used these quadratic regressions to interpolate the values.

## Typographical Comments

Dr. King is wrong in asserting that there is a decimal point error in Table 6. Contrary to Dr. King’s comment, the injury risk analysis utilized in the STAR system is not dependent on impact location. If Dr. King was confused, he could have asked for clarification, or referenced any of the material on our website that we developed to help people understand and recreate the STAR system.^24^

Dr. King is wrong in asserting that the STAR system is flawed because he was not able to reproduce the injury risk curve. The STAR system utilizes an injury risk curve based on collegiate football concussion incidence rates. The collegiate coefficients in Table 5 of the STAR paper for the risk curve are correct, and simply need to be plugged into Eq. (6) of the STAR paper. Several other researchers have been able to recreate and use this injury risk curve from the paper.^11^,^29^ The coefficients provided in Table 5 have been plugged into Eq. (6) to form the injury risk curve used by the STAR system below:

6 $$ R(a) = \frac{1}{{1 + e^{ - ( - 9.805 + 0.051x)} }} $$

The second injury risk curve based on professional football concussion incidence rates was provided as an illustrative example, and does not affect the STAR system. With that said, there was a typographical error in Table 5 of the STAR paper. The β coefficient for professional football should be written as 0.0497, rather than 0.497. This addresses Dr. King’s comment about re-creating Fig. 6.

We thank Dr. King for bringing to the readers’ attention to an error in Eq. (4) in the published manuscript. During the journal review process, the regression analysis was changed from solving for drop height as a function of head acceleration to solving for acceleration as a function of drop height. Unfortunately, Eq. (4) in the STAR paper was not updated to reflect this. Equation (4) should be written as:

4 $$ a = p_{1} H^{2} + p_{2} H + p_{3} $$

With this correction, Dr. King’s comments related to acceleration and drop height should be resolved. This correction does not affect any of the results in the STAR paper as the correct values were used.

## Conclusion

Dr. King’s conclusions are not supported by any peer reviewed data or findings. The STAR system is the culmination of a decade’s worth of research, and is based on two fundamental principles: (1) helmets that better manage the impact energy by resulting in lower head accelerations will reduce the risk of concussion (2) each test condition is weighted so that they represent how often each impact scenario is experienced on the field by players.

In summary, we return back to the question that we started with: do you want to buy a helmet that lowers head acceleration? The STAR system uses current scientific knowledge to illustrate which helmets are better at reducing head acceleration. We will continue to purchase the best helmets for the Virginia Tech football team based on the STAR system.
